# Biomarkers of food intake for cocoa and liquorice (products): a systematic review

**DOI:** 10.1186/s12263-018-0610-x

**Published:** 2018-07-27

**Authors:** Charlotte C. J. R. Michielsen, Enrique Almanza-Aguilera, Elske M. Brouwer-Brolsma, Mireia Urpi-Sarda, Lydia A. Afman

**Affiliations:** 1 Division of Human Nutrition and Health, Wageningen University and Research Centre, Stippeneng 4, 6708 WE Wageningen, The Netherlands; 2 Department of Nutrition, Food Sciences and Gastronomy, Biomarkers and Nutrimetabolomics Laboratory, XaRTA, INSA-UB, Faculty of Pharmacy and Food Sciences, University of Barcelona, Barcelona, Spain; 3 CIBER Fragilidad y Envejecimiento Saludable (CIBERFES), Instituto de Salud Carlos III, 08028 Barcelona, Spain

**Keywords:** Licorice, Liquorice, Cocoa, Cacao, Chocolate, Metabolites, Metabolomics, Biomarkers

## Abstract

**Background:**

To unravel true links between diet and health, it is important that dietary exposure is accurately measured. Currently, mainly self-reporting methods (e.g. food frequency questionnaires and 24-h recalls) are used to assess food intake in epidemiological studies. However, these traditional instruments are subjective measures and contain well-known biases. Especially, estimating the intake of the group of confectionary products, such as products containing cocoa and liquorice, remains a challenge. The use biomarkers of food intake (BFIs) may provide a more objective measurement. However, an overview of current candidate biomarkers and their validity is missing for both cocoa- and liquorice-containing foods.

**Objective:**

The purpose of the current study was to (1) identify currently described candidate BFIs for cocoa (products) and liquorice, (2) to evaluate the validity of these identified candidate BFIs and (3) to address further validation and/or identification work to be done.

**Methods:**

This systematic review was based on a comprehensive literature search of three databases (PubMed, Scopus and ISI web of Science), to identify candidate BFIs. Via a second search step in the Human Metabolome Database (HMDB), the Food Database (FooDB) and Phenol-Explorer, the specificity of the candidate BFIs was evaluated, followed by an evaluation of the validity of the specific candidate BFIs, via pre-defined criteria.

**Results:**

In total, 37 papers were included for cocoa and 8 papers for liquorice. For cocoa, 164 unique candidate BFIs were obtained, and for liquorice, four were identified in total. Despite the high number of identified BFIs for cocoa, none of the metabolites was specific. Therefore, the validity of these compounds was not further examined. For liquorice intake, 18-glycyrrhetinic acid (18-GA) was found to have the highest assumed validity.

**Conclusions:**

For cocoa, specific BFIs were missing, mainly because the individual BFIs were also found in foods having a similar composition, such as tea (polyphenols) or coffee (caffeine). However, a combination of individual BFIs might lead to discriminating profiles between cocoa (products) and foods with a similar composition. Therefore, studies directly comparing the consumption of cocoa to these similar products are needed, enabling efforts to find a unique profile per product. For liquorice, we identified 18-GA as a promising BFI; however, important information on its validity is missing; thus, more research is necessary. Our findings indicate a need for more studies to determine acceptable BFIs for both cocoa and liquorice.

**Electronic supplementary material:**

The online version of this article (10.1186/s12263-018-0610-x) contains supplementary material, which is available to authorized users.

## Background

Several epidemiological studies have observed relationships between habitual intake of cocoa (products) and liquorice and health. Beneficial effects associated to the consumption of cocoa and cocoa product intake include a positive association with flow-mediated vasodilatation and inverse associations with blood pressure, serum insulin, HOMA-IR, calcified atherosclerotic plaques in the coronary arteries, incident cardiovascular diseases, cardiac mortality, cardiovascular mortality and all-cause mortality [1–6]. Liquorice has been widely exploited for medicinal purposes including its use as remedy in case of sore throat or cough [7, 8]. However, even though beneficial effects have been reported for both the consumption of cocoa- and liquorice-containing products, many of these products are often energy-dense foods, high in sugar and fats. Therefore, intake of these products in high amounts is not recommended, as it is associated with obesity and related diseases [9, 10]. Also, contraindications for the use of liquorice in large amounts have been reported in specific conditions, especially during pregnancy and in patients with hypertension, hypokalaemia or hepatic or kidney failure [11, 12].

To unravel true links between diet and health in epidemiology studies, it is essential to accurately assess dietary exposure. Currently, the use of self-reporting methods, such as 24-h dietary recalls, food diaries, and food-frequency questionnaires are the most frequently used instruments to assess food exposure in epidemiological studies. However, these conventional methods are subjective measures; they contain well-known biases, such as reporting and recall biases [13, 14], and do not take into account individual characteristics and differential metabolic responses to the intake of different food components and food bioactives. Therefore, the observed relationships between nutrition and health could have been affected, thereby possibly leading to inconsistencies in the field of nutritional research. In particular, accurate estimation of the intake of cocoa- and liquorice-containing products is difficult. This is partly due to the fact that the moments on which these types of foods are consumed are often not planned. Furthermore, people have difficulties determining accurate portion sizes, they might underreport their intake of these types of food as they are believed to be unhealthy, and they might not be able to recall all the foods that contain these types of compounds. Hence, there is an urgent need for more accurate measurements of food intake, especially for cocoa- and liquorice-containing foods. As a result of the application of metabolomics in the nutrition field, candidate biomarkers of food intake (BFIs) are increasingly described in the literature [15]. BFIs are objective measures of actual food intake and as such could be used in conjunction with the conventional methods, to improve the quality of dietary assessment in nutritional science and to assist in examining true associations between nutrition and health [16]. In the literature, many different biomarker classification schemes exist [17, 18]. In this manuscript, we defined BFIs according to the definition proposed by Gao et al. [18]. In short, we included all biomarkers described in the literature after the intake of cocoa products, liquorice products or their components that can be used to estimate recent or average intakes of these entities. Since cocoa and cocoa products are consumed all over the world, the number of potential BFIs for cocoa (products) is rapidly rising in the literature. Liquorice is also widely consumed, especially in Europe, and can be used as an ingredient in different food products. However, potential BFIs for the consumption of liquorice (products) are less well explored [19]. Currently, three approaches are used for BFI identification, namely, (1) acute or chronic intervention studies where metabolic profiles are examined after a specific load of the food of interest, (2) dietary pattern studies where metabolic profiles are examined after subjects adhere to a certain dietary pattern and (3) observational studies where metabolic profiles are compared between consumers and non-consumers of a specific food of interest [20, 21]. To examine the validity of a candidate BFI, it is important that the BFI has been evaluated using all of these approaches. An overview of already identified candidate BFIs for cocoa (products) and liquorice, as well as an evaluation of their validity, is needed to identify known and accepted BFIs, as well as to identify what information is still missing and requires further investigation. Hence, the aims of this systematic review were (1) to identify currently described candidate BFIs for cocoa (products) and liquorice in the literature, (2) to evaluate the validity of these identified candidate BFIs and (3) to address further identification and/or validation work to be done. This systematic review is performed in the frame of the FoodBAll (Food Biomarkers Alliance) project under the Joint Programming Initiative—A Healthy Diet for a Healthy Life (JPI-HDHL) (http://www.foodmetabolome.org/).

## Materials and methods

### Identification of biomarkers

**Fig. 1:**
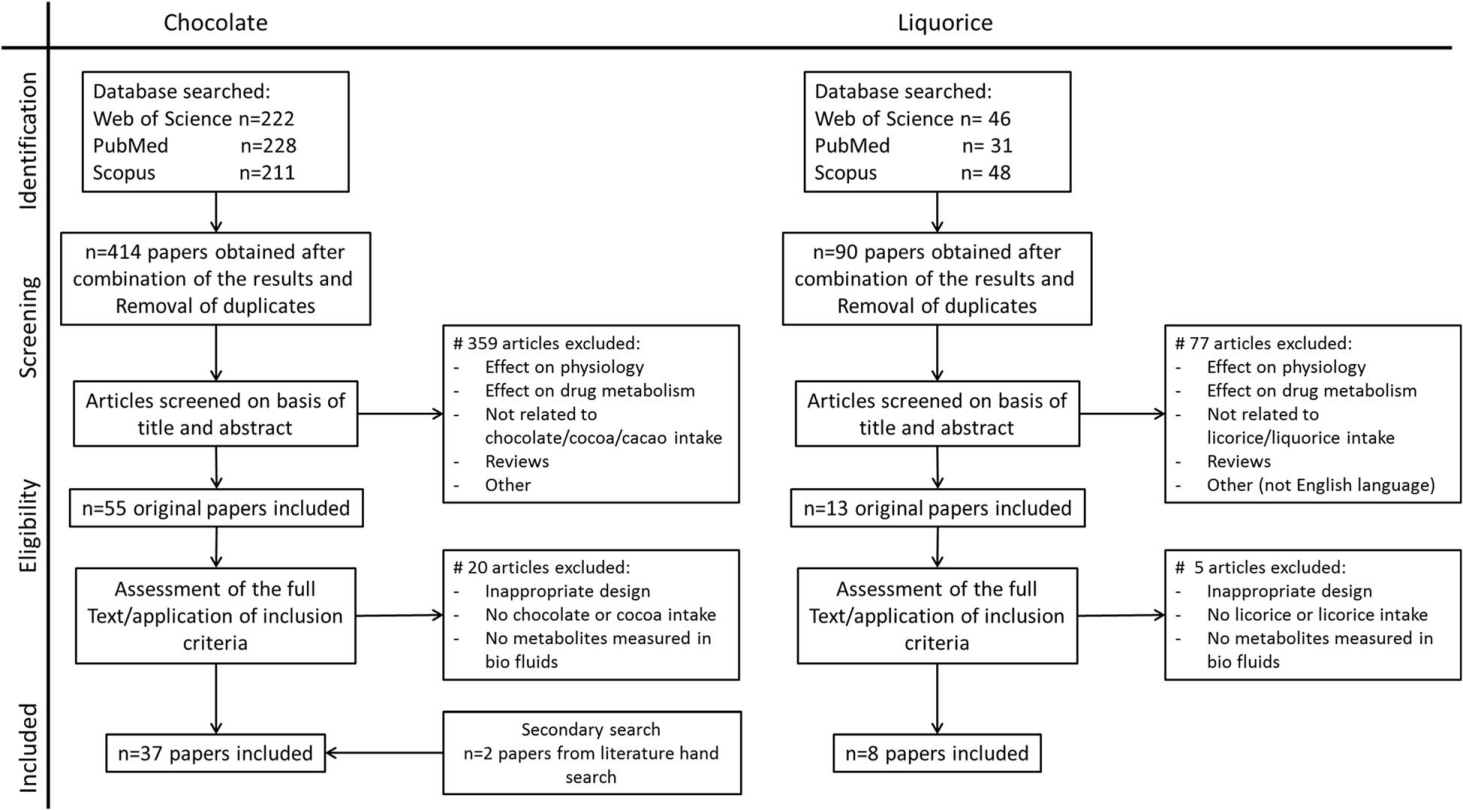
Flowchart selection of papers for biomarkers of chocolate and liquorice intake. Searches for 440 cocoa and liquorice biomarkers were done up to October 2016 and March 2017, respectively

In order to identify papers on BFIs for both cocoa and liquorice, we carried out an extensive literature search following the Biomarker of Food Intake Reviews (BFIRev) methodology proposed previously [22]. In short, for this systematic review, all elements of the PRISMA statement [23] relevant for a literature search on biomarkers were used. Original papers and reviews were searched in Scopus, PubMed and ISI Web of Knowledge, using the grouped search terms (biomarker* OR marker* OR metabolite* OR biokinetics OR kinetic* OR biotransformation) AND (human* OR men OR women OR patient* OR volunteer* OR participant*) AND (trial OR experiment OR study OR intervention) AND (urine OR plasma OR serum OR blood OR excretion) AND (intake OR meal OR diet OR ingestion OR consumption OR eating OR drink* OR administration). For the BFI for cocoa, AND (cocoa* OR chocolate* OR cacao* OR Theobroma) was added, and for the BFI for liquorice, AND (liquorice OR liquorice) was added. Three independent reviewers (CCJRM, EAA and EMB-B) selected the papers in a process outlined in Fig. 1. Only English papers were included, and no restriction was applied for the publication dates (searches for cocoa and liquorice biomarkers were done up to October 2016 and March 2017, respectively). Papers describing the effect on physiology, bio stability and/or drug metabolism were excluded. Furthermore, we excluded papers that examined chocolate products without cocoa (for example white chocolate) and liquorice products without liquorice root extract (for example red liquorice). Animal studies were also excluded, since it remains to be determined whether animal models are valid models for examining the absorption, distribution, metabolism and excretion of compounds in humans [24]. Initially, only titles and abstracts were screened to determine if they satisfied the selection criteria. Any disagreements between the reviewers were resolved through consultation. Next, full-text papers were retrieved for the selected titles. Additional papers were identified from reference lists of the retrieved papers and from the selected book chapters or reviews, called hand searches. Again, three independent reviewers (CCJRM, EAA and EMB-B) assessed the obtained papers to ensure that they were in agreement with the inclusion criteria. A data collection form was designed to streamline the process of extracting relevant information from the selected studies. This form contained the following items: dietary factor, dose of intervention, study design, number of subjects, analytical method, sample type, discriminating metabolites (BFIs), notes and primary reference(s).

### Specificity evaluation of the identified biomarkers

To evaluate the apparent specificity of each of the identified candidate BFI, a second search step consisting of two parts was performed. Firstly, the compound databases HMDB (http://www.hmdb.ca/), FooDB (http://foodb.ca/) and Phenol-Explorer (http://phenol-explorer.eu/) were used to screen the identified biomarkers of food intake. If a compound was found to be present in non-cocoa- or non-liquorice-related foods, it was removed from the selection. Secondly, an additional search was performed for the remaining selection, using combinations of the grouped search terms (“the name and synonyms of the compound”) AND (biomarker* OR marker* OR metabolite* OR biokinetics OR biotransformation) AND (trial OR experiment OR study OR intervention) AND (human* OR men OR women OR patient* OR volunteer* OR participant*) AND (urine OR plasma OR serum OR blood OR excretion) AND (intake OR meal OR diet OR ingestion OR consumption OR eating OR drink* OR administration) in any of the listed databases above or Google Scholar. If the compound was found to be present in non-cocoa- or non-liquorice-related foods in this second search, it was removed from the selection.

### Validity of the identified biomarkers

For the final selection of identified BFIs, the validity was assessed. For this, we have used the method proposed by Dragsted et al. [25]. An elaborate explanation on how the validity was assessed, including the pre-defined criteria; how the validity questions were constructed; and a discussion on the strengths and weaknesses of this method can be found in the paper of Dragsted and colleagues [25]. In short, the validity was assessed via answering nine questions, where possible answers were Yes (Y), No (N) or unknown/uncertain (U) where appropriate. If Y was answered to a question, this would increase the assumed validity of that specific biomarker. The questions taken into consideration were related to current knowledge about biological, analytical and nutritional aspects of the methodology and are based on a thorough search of previous literature. The questions were as follows: (1) Is the marker compound plausible as a specific BFI for the food or food group (chemical/biological plausibility)? (2) Is there a dose-response relationship at relevant intake levels of the targeted food (quantitative aspect)? (3) Is the single-meal time-response relationship described adequately to make a wise choice of sample type and time window (single-dose kinetics)? (4) Is the biomarker kinetics for repeated intakes of the food/food group described adequately providing the frequency of sampling needed to assess habitual intake (e.g. the cumulative aspects: does the biomarker accumulate in the body over time after repeated intakes?)? (5) Has the marker been shown to be robust after intake of complex meals reflecting dietary habits of the targeted population (robustness)? (6) Has the marker been shown to compare well with other markers or questionnaire data for the same food/food group (reliability)? (7) Is the marker chemically and biologically stable during bio specimen collection and storage, making measurements reliable and feasible? (8) Are analytical variability (CV%), accuracy, sensitivity and specificity known as adequate for at least one reported analytical method? (9) Has the analysis been successfully reproduced in another laboratory (reproducibility)? In the end, the number of times a Y was given per biomarker was added, in order to get insight in the validity of a selected biomarker. The higher this number, the more is known about the compound, the higher its assumed validity. This score will therefore reflect the current level of validity of that particular compound and pinpoints what additional research is needed to increase the validity of that particular compound.

## Results

### Candidate BFIs, identification

#### Cocoa (products) BFIs

A total of 414 potentially relevant papers were identified from searches in PubMed, Web of Science and Scopus. After a first screening of the title and abstract, 55 papers were collected as full text and assessed for further inclusion. Then, 20 papers were excluded due to either inappropriate study designs (e.g. animal studies), unreported cocoa or chocolate intakes or unreported metabolites/markers. Finally, two additional papers were identified via hand searches and added to the list, which lead to a total inclusion of 37 papers (Fig. 1). Intervention studies (*n* = 34) were the most frequently employed methods to determine candidate BFIs for cocoa (products) (Additional file 1: Table S1). In these intervention studies, the participants consumed the following: cocoa-based beverages (*n* = 20), chocolate (*n* = 8), a mixture of chocolate and cocoa beverages (*n* = 2), cocoa-based nut cream and/or polyphenol capsules (*n* = 2) and cocoa extract as part of a ready-to-eat meal (*n* = 1). Of the 34 intervention studies, there were 16 acute crossover studies, 12 acute single-dose studies, 3 crossover intervention studies (ranging from 4 to 6 weeks), 3 parallel intervention studies (ranging from 5 days to 6 months) and 2 single-arm intervention studies (4 and 12 weeks). Only 2 observational studies [26, 27] using estimated self-reported dietary intake data reported candidate BFIs for cocoa and cocoa-product consumption. The most commonly used bio samples were urine (*n* = 26) and plasma (*n* = 18). Urine was collected mainly as 24-h urine (*n* = 16), but also as spot urine (*n* = 7), as 8-h urine (*n* = 1) and as 72-h urine (*n* = 2). Number of subjects ranged from 1 up to 59 in the intervention studies and up to 481 for the cross-sectional study. Furthermore, 28 studies used a targeted approach to determine candidate BFIs, and 10 studies used an untargeted approach, by which more compounds could be identified. Among the selected papers, a total of 164 different compounds were found as candidate BFIs for cocoa (products) intake (Additional file 1: Table S1). (±)-Catechin and (−)-epicatechin derivatives were by far the group of metabolites most reported after cocoa intake, followed by hydroxyphenylvalerolactones, hydroxyphenylvaleric acids and methylxanthines.

#### Liquorice (products) BFIs

A total of 90 potentially relevant papers were identified from searches in PubMed, Web of Science and Scopus. After a first screening of the title and the abstract, 13 papers were collected as full text and assessed for further inclusion. Then, 5 papers were excluded due to either inappropriate study designs (e.g. animal studies), unreported liquorice intakes or unreported metabolites/markers. No additional papers were obtained via hand searches, leading to a total inclusion of 8 papers (Fig. 1). All the included studies were intervention studies to determine candidate BFIs for liquorice (products) (Table 1). In these intervention studies, the participants consumed isolated compounds of liquorice (*n* = 4), solid liquorice (*n* = 2) and liquid liquorice (*n* = 1) or consumed a mixture of a compound or solid liquorice (*n* = 1). In the eight papers, there were four acute single-dose studies, two acute crossover studies, two parallel single-dose studies, two parallel placebo-controlled intervention studies (1 and 4 weeks) and one parallel intervention study (5 days). No observational study using estimated self-reported dietary intake data reported candidate BFI liquorice (product) consumption nor were there any longer term studies performed, as the longest study was 4 weeks. Again, plasma and urine were the most commonly used bio samples, *n* = 7 and *n* = 4, respectively. Multiple spot urine collections were used in two studies, prolonged collection of urine (for 4 and 5 days) was used in two other studies, and one study collected 24-h urine. The number of subjects ranged from 1 up to 60. Furthermore, all studies used a targeted approach to determine candidate BFIs. Among the selected papers, a total of four different compounds were found as candidate BFIs for liquorice (products) intake (Table 1), namely, 18-glycyrrhetinic acid (18-GA), 18-GA glucuronides, 3β-monoglucuronyl-18β-glycyrrhetinic acid (3-MGA) and glabridin.

**Table 1:** List of reported candidate liquorice biomarkers of intake, including information about dosage, study design, number of subjects, method used, sample type and the primary references

Dietary factor		Dose of intervention	Comparable to no. of grams of liquorice containing 0.17% GL^a^	Study design	Number of subjects (no. of men)	Age range (years)	BMI range^b^ (kg/m^2^)	Analytical method	Approach	Sample type and time	Candidate biomarkers of food intake	HMDB ID*	Primary ref.
Liquorice (products)													
Solid	Katzen Kinder by Katjes (0.05% GL)	200 g	135	Acute single-dose study	4 (1)	26–29	18.6–28.6	LC-MS/MS (LC-ESI-MS)	Targeted	Blood (0, 0:05, 1, 2, 3, 5 and 7 h) Urine (regularly)	Blood, 18-GA (highest after 6 h; 150–434 ng/ml) Urine −	HMDB0011628	[53]
	Solid liquorice (0.23% GL)	50, 100 and 200 g daily for 5 days 500 g within 4 h	68, 135 and 271 676	Parallel intervention study (5 days) Acute single-dose study	3 (0) 1 (1)	19–23 22	U U	GC-MS	Targeted	Urine (all up to 5 days)	Urine, 18-GA		[52]
Isolated compounds	Glycyrrhizin (GL)	600 mg in 2 dl water (excretion study)	353	Acute single-dose study	6 (5)	24–40	U	HPLC-UV	Targeted	Urine (all up to 4 days)	18-GA 3-MGA (Cmax 0.49–2.69 μg/ml)	HMDB0037827	[99]
	Glycyrrhetinic acid (GA)	500 mg	509^c^	Acute single-dose study	10 (10)	24–38	U	LC analyser	Targeted	Serum (0, 2, 4, 7, 10 and 24 h) Urine (0, 2, 4, 7, 10 and 24 h)	Serum, 18-GA (max after 2–4 h 13.4 μmol/l) Urine −		[54]
	Liquorice extract (LE) vs. glycyrrhizin	21 g LE vs. 1600 mg GL	941	Acute crossover study	16 (8)	U	U	HPLC-UV	Targeted	Plasma (up to 36 h)	18-GA (both after LE and GL consumption)		[55]
	Glycyrrhetinic acid	500, 1000 and 1500 mg with water	294, 588 and 882	Parallel single-dose study	6 (6)	27–31	H, 176–180 cm W, 65–73 kg	HPLC	Targeted	Plasma (− 0.5 h, every 30 min up to 8, 9, 10 and 12 h; 1000 mg additionally at 14 and 24 h; 1500 mg additionally at 48 h) Urine (24 h)	Plasma, 18-GA (Cmax; 500, 4.5 mg/l; 1000, 7 mg/l; 1500, 9 mg/l, tmax 3-4 h) Urine, 18-GA, and 18-GA glucuronides (only at 1500 mg dose)	18-GA glucuronides: not in HMDB	[51]
Liquid	Liquorice flavonoid oil (LFO, 90% MCT, 8% polyphenols, 1% glabridin)	300, 600 and 1200 mg of LFO vs. placebo	–	Parallel single-dose study Parallel placebo-controlled intervention study (1 week) Parallel placebo-controlled intervention study (4 weeks)	15 (15) 42 (21) 60 (30)	20–60	BW > 50-kg men > 40-kg women	SPE-LC-MS/MS	Targeted	Plasma (0, 2, 4, 6, 8 and 24 h) Plasma (0, 4 and 24 h on day 1 and day 7) Plasma (0 h on days 1, 14 and 28)	Glabridin (Cmax at 4 h, around 0.8–2.1 ng/ml depending on the dose)	HMDB0034188	[56]
Mixture	Glycyrrhetic acid, glycyrrhizin and solid liquorice (0.15% GL)	130 mg 18-GA^c^ in water-propyleneglycol 225 mg GL in water 150-g sweet liquorice 150-g salted liquorice	132	4-way random acute crossover study	16 (8)	U	U	HPLC	Targeted	Plasma (− 1, 2, 2.5, 4, 5.5, 7, 8.5, 10, 11.5, 13, 14.5, 18, 22, 32, 48 and 56 h)	18-GA (Cmax about 1000 μg/l, same after salty or sweet-tasting liquorice)		[50]

^a^Calculated based on the report of the European Scientific Committee on Food, reporting a mean glycyrrhizin content in liquorice confectionery of 0.17% [96]. This is not calculated for LE and LFO, as it is unclear what the exact percentages of these compounds are in liquorice itself

^b^If BMI was reported in the article, only height and/or weight were reported. If nothing was reported at all, we have written down U

^c^Amount GA was first converted to amount GL, as 130 mg GA is equivalent to 225 mg GL, according to [50]

*HMDB-ID is only reported once for the same candidate biomarker of food intake

*18-GA* 18(β)-glycyrrhetic acid or 18(β)-glycyrrhetinic acid, *3-MGA* 3β-monoglucuronyl-18β-glycyrrhetinic acid, *BW* body weight, *GC-MS* gas chromatography-mass spectrometry, *GL* glycyrrhizin, *H* height, *HPLC* high-performance liquid chromatography, *LC-ESI-MS* liquid chromatography-electrospray ionization-tandem mass spectrometry, *LC-MS/MS* liquid chromatography-tandem mass spectrometry, *LE* liquorice extract, *LFO* liquorice flavonoid oil, *Ref.* reference, *SPE-LC-MS/MS* solid phase extraction liquid chromatography tandem mass spectrometry, *U* unknown: values not reported in the article, *UV* ultraviolet, *W* weight

### Specificity and validity of the identified BFIs

#### Cocoa (products) BFIs

The specificity of each of the identified candidate BFI was evaluated via a second search step. First, the 164 candidate BFIs were screened for specificity for cocoa or cocoa products in the compound databases HMDB, FooDB and Phenol-Explorer. Based on the presence in other foods, 63 markers were removed from the selection. Three turned out to have an unclear formulation (e.g. missing information on the place of side chains) and were therefore excluded. Based on the search results in the compound databases, the following metabolites were removed because of their presence in other food: epicatechins, catechins and vanillic acid. Important to note is that most of their biotransformation products (e.g. glucuronides and sulfates), a total of 27 metabolites, were not found in the compound databases. As it is known that these biotransformation products are all related to epicatechins, catechins or vannilic acid, they were also removed from the selection. Furthermore, 11 metabolites of caffeine (theobromine, paraxanthine, theophylline, and their biotransformation products) were removed from the selection, since these metabolites are produced in the gut microbiota upon intake of food products containing caffeine, such as coffee, tea or cola (source: HMDB), and they were therefore suspected not to be specific BFIs for chocolate. The specificity of the remaining 61 candidate BFIs was further examined by performing additional searches in Scopus, ISI Web of Science, PubMed or Google Scholar, following the syntax explained in the “Materials and methods” section. Hydroxyphenylvaleric acids and hydroxyphenylvalerolactones were found to be microbial-derived metabolites from polyphenol intake (flavan-3-ols, flavonols and flavanones) [28–30] and were therefore suspected not to be specific BFIs for chocolate intake but markers for all polyphenol-containing foods, such as almonds or tea [28] (removal of 30 metabolites). *N*-Phenylpropenoyl-l-amino acids are known to be particularly common not only in cocoa but also in coffee and other plant-based foods such as red clover [31, 32] and were therefore removed from the selection (removal of 13 metabolites). Furthermore, a ketone body (3-hydroxybutyrate) and other endogenous metabolites (tyrosine sulfate, *N*-methylguanine, methylglutarylcarnitine, guanidinoacetate) were removed from the selection [27, 33–35]. And lastly, metabolites related to protein intake (xanthurenic acid, indoxyl-sulfate, 4-cresol sulfate [36, 37]), vegetable intake (phenylacetylglutamine [38, 39]), coffee intake (furoylglycine [40]), tea intake (cyclo(Ser-Tyr), cyclo(Pro-Pro) [41]), beer intake (cyclo(Propylalanyl), cyclo(Pro-Pro) [42]), aspartame intake (cyclo(Aspartyl-Phenylalanyl) [43]), nicotinic acid metabolism (hydroxynicotinic acid [44]) and food packaging (di-iso-nonyl phthalate, di-(2-ethylhexyl)phthalate [45, 46]) were also removed from the selection. This meant that none of the currently identified BFIs for cocoa (products) made the final selection, and therefore, the validity was not checked for any of the candidate BFIs.

#### Liquorice (products) BFIs

The specificity of each of the identified candidate BFI for liquorice (products) was also evaluated via a second search step. First, the four candidate BFIs were screened for specificity for liquorice (products) in the compound databases HMDB, FooDB and Phenol-Explorer. The compound 18-GA glucuronide could not be found in any of the compound databases. However, it is known to be the product of the hepatic metabolism of 18-GA [47], and therefore, it was excluded from further evaluation. The other three compounds were found in the databases HMDB and FooDB and were linked to the presence in herbs/spices (18-GA, glabridin) or were known as a sweetener (3-MGA). 3-MGA is also a metabolite of GA and is known to be excreted via urine in small quantities and to be a possible biomarker for liquorice-induced adverse effects [19, 48]. Therefore, we decided to include 3-MGA for further evaluation. In Table 1, an overview is presented of the studies on liquorice BFIs, including details on dose used in the study, number of subjects included, which analytical method was used to analyse the samples, which BFIs were found and the primary reference. The specificity of all three candidate BFIs was further examined by performing additional searches in Scopus, ISI Web of Science, PubMed or Google Scholar, which gave us no indication that these metabolites were related to the intake of other food products. On the basis thereof, and on the eight originally included papers, the validity of the three selected candidate BFIs for liquorice (products) was evaluated (Table 2). As can be seen in Table 2, the compound 18-GA in urine after liquorice (product) consumption had the highest assumed validity; however, uncertainty still remains about its specificity, since liquorice root extract itself is used in many other products, such as chewing gum, other confectionary or beverages (question 1) [19, 47, 49, 50]. Krahenbuhl et al. [51] observed a clear dose-response relationship for 18-GA in plasma; however, the concentration GA used in this study was comparable to an intake of liquorice ranging from 1.7 to 5.2 kg; therefore, no relevant intake levels of the targeted food were examined in this study. In the study of Kerstens et al. [52], 50–200 g of liquorice were consumed, showing a dose-response relationship of 18-GA in urine (question 2). 18-GA was found to be traceable in urine after the intake of 50 g of solid liquorice, and around 0.04% of the total intake could be traced back after 51 h in total [52]. A peak in 18-GA concentration was measured after 6 h in the blood after the consumption of 200 g of solid liquorice [53], or between 1.5 and 40 h in urine after the consumption of 600 mg of the compound glycyrrhizin (content comparable to 353 g of liquorice, Table 1) [19]. 18-GA was also traceable in 24-h urine after the consumption of 1500 mg of the compound GA (content comparable to 882 g of liquorice, Table 1) [51]. In serum, a peak was observed after 2–4 h of the consumption of 500 mg of the compound GA [54], and in plasma, the peak time was after 3–4 h after the consumption of 21 g of liquorice extract [55], or 500 mg and higher concentrations of the compound GA [51]. Best sampling time for 3-MGA in urine was unknown, since time to maximum peak height ranged from 1.5 to 39.5 h. This high interindividual variability in time to maximum peak height was likely caused by differences in metabolism rate and enterohepatic cycling of the compound [19] (question 3). None of the studies examined repeated intakes of liquorice, or isolated compounds of liquorice roots (question 4). In plasma, serum and urine, 18-GA was measured after subjects continued with their habitual diet during the measurements (question 5) [50–55]. Since none of the studies was an observational study, or included questionnaire data, the answers to question 6 were “no” for all candidate BFIs. Kerstens et al. [52] did examine whether 18-GA could be used to detect whether two patients had consumed liquorice, even if the patients denied having eaten liquorice-containing products. They observed that it was indeed possible to detect liquorice intake via measuring 18-GA in urine, after which the patient indeed admitted to have eaten liquorice containing products. Unfortunately, data on actual liquorice intake (via for example food frequency questionnaires or 24-h recalls) were not recorded in this study. Question 7 concerned the chemical and biological stability during specimen collection and storage. In the eight included papers, it was described that urine samples were stored at 4 °C [19, 54] or − 20 °C [52, 53], and blood, plasma and serum samples were stored at − 20 °C [50–53, 55] before continuing with the analyses. In some cases, no information was available about the storage temperature of the samples [56], and for all eight studies, it was unclear how long the samples had been stored. Analytical variability for measuring 18-GA in plasma was 3% [51]; in urine, CV% was between 6 and 9.3% [51, 53]; in serum, there was a 2.1% within assay variability and 8.5% between assay variability [54]; and in blood, CV% was between 4.6 and 6.3% [53]. For 3-MGA, the repeatability of the measurements within and between days was tested, with peak height 4.88 and 7.21 RSD%, respectively, and peak area 2.61 and 6.21 RSD%, respectively [19]. In the papers of Raggi et al. [55], Aoki et al. [56] and Ploeger et al. [50], no details about analytical variability were presented (question 8). Lastly, none of the analyses described in the eight selected papers were reproduced by another laboratory. The analyses used were either described in the paper for the first time [19, 50, 52, 53, 55] or had previously been performed at their own lab [51, 54, 56] (question 9).

**Table 2:** Evaluation of the validity of the identified candidate biomarkers of food intake for liquorice

Metabolite	Bio fluid	Q1	Q2	Q3	Q4	Q5	Q6	Q7	Q8	Q9	Sum	References
Candidate liquorice biomarkers of food intake												
18-glycyrrhetic acid	Plasma	U	U	Y	U	Y	N	U	Y	N	3	[50, 51, 55]
	Serum	U	U	Y	U	Y	N	U	Y	N	3	[54]
	Blood	U	U	Y	U	N	N	U	Y	N	2	[53]
	Urine	U	Y	Y	U	Y	N	U	Y	N	4	[51–53]
3β-monoglucuronyl-18β-glycyrrhetinic acid	Urine	U	U	N	U	Y	N	U	Y	N	2	[19]
Glabridin	Plasma	U	U	U	U	N	N	U	N	N	0	[56]

*Y* yes, *N* no, *U* unknown

Q1: Is the marker compound plausible as a specific BFI for the food or food group (chemical/biological plausibility)?

Q2: Is there a dose-response relationship at relevant intake levels of the targeted food (quantitative aspect)?

Q3: Is the single-meal time-response relationship described adequately to make a wise choice of sample type and time window (single-dose kinetics)?

Q4: Is the biomarker kinetics for repeated intakes of the food/food group described adequately providing the frequency of sampling needed to assess habitual intake (e.g. cumulative aspects)?

Q5: Has the marker been shown to be robust after intake of complex meals reflecting dietary habits of the targeted population (robustness)?

Q6: Has the marker been shown to compare well with other markers or questionnaire data for the same food/food group (reliability)?

Q7: Is the marker chemically and biologically stable during bio specimen collection and storage, making measurements reliable and feasible?

Q8: Are analytical variability (CV%), accuracy, sensitivity and specificity known as adequate for at least one reported analytical method?

Q9: Has the analysis been successfully reproduced in another laboratory (reproducibility)?

## Discussion

The present systematic review examined the current status of candidate BFIs for the consumption of cocoa (products) and liquorice. In total, 37 relevant papers were included for cocoa (products) [26, 27, 32–34, 44, 57–87], and 8 relevant papers were included for liquorice (products) [19, 50–56]. For cocoa (products), 164 different compounds were identified as candidate BFIs in the 37 obtained papers. After evaluating the specificity of these compounds, none of these candidate BFIs turned out to be specific for cocoa (products). Therefore, the validity of these compounds was not further examined. For liquorice (products), 4 different compounds were identified as candidate BFIs in the 8 obtained papers. After evaluating the specificity and the validity of these 4 compounds, 18-GA in urine was found to have the highest assumed validity.

Regarding the 164 cocoa (products) BFIs (Additional file 1: Table S1), none of the identified compounds was specific. Our results indicated that most of the identified BFIs for cocoa (products) were also found or are expected to be found after the consumption of foods such as tea, coffee or red wine. This is due to similarities in the composition of these foods, such as a high polyphenol content or the presence of caffeine compounds [88–91]. Only a few randomised trials (both short and long term) have compared the metabolite profile after consumption of cocoa (products) with other products showing a similar metabolite profile, such as the metabolite profiles observed after tea [63, 71] or coffee [63]. Up till now, no metabolites were obtained that could discriminate between these food products. However, only a small amount of metabolites was measured in these studies via targeted approaches. With untargeted approaches, a higher number of compounds can be measured in bio fluids and therefore might elucidate compounds that are specific and valid BFIs for cocoa (products). Before any conclusion can be drawn about the specificity of the identified BFIS for cocoa (products), more randomized intervention studies comparing food products with a similar metabolite excretion pattern, using untargeted approaches, are needed. Furthermore, it remains to be explored whether the dose-response relationship is equal after consuming for example same amounts of tea and a cocoa drink [63]. Another important point to consider is that the way of processing of the cocoa beans, the cocoa variety and the origin of the cocoa bean can affect the final concentration of compounds in the cocoa product [92]. In addition, cocoa beans are subject to seasonal variation, again affecting concentrations of several compounds in the beans [93, 94]. In this review, we focused on single BFIs for cocoa intake, which turned out to be nonspecific. A combination of several biomarkers, a so-called biomarker profile or panel of biomarkers might however increase the specificity for cocoa intake. Garcia-Aloy and colleagues [95] have examined this possibility in an untargeted study and found a combined model for cocoa consumption that included 7-methylxanthine and dihydroxyphenylvalerolactone glucuronide. This combined model was a better discriminant for cocoa consumption compared to all individual metabolites. It is essential to explore whether this combination of compounds will also discriminate cocoa (products) intake from tea intake or coffee intake.

The four identified liquorice BFIs (Table 1), 18-GA, 18-GA glucuronides, 3-MGA and glabridin, were only described in relation to liquorice intake or liquorice root extracts. However, liquorice root extracts are known to be used in a variety of products, such as different sweets, chewing gums, chewing tobacco and tea, or even in (alcoholic) drinks and medicinal products, often as a sweetening or flavouring agent [19, 47, 49, 50]. It will therefore be necessary to examine the relative content and contribution of liquorice extract in these products and in the habitual consumed diets overall. An important point to consider is that the content of liquorice root extracts may vary from product to product, depending on the characteristics the producer desires for that product, which causes variation in the final concentrations of liquorice root extracts in these products. For example, in the solid liquorice that was consumed in the included studies, the GL content varied from 0.05 up to 0.23%. 18-GA in urine was the most promising and most studied candidate BFI out of the three candidates for liquorice (products). To increase its validity, studies on repeated intake, habitual food consumption, dietary patterns, stability during storage and reproducibility of the methods between labs are still needed. Important to note is that in all studies examining liquorice biomarkers, subjects consumed 68 g of liquorice or more (when standardized to the average glycyrrhizin content in liquorice confectionary of 0.17% [96], this is equivalent to an intake of GL of 116 mg), while the European Scientific Committee on Food advises that ingestion of liquorice should not exceed 58 g per day (equivalent to an intake of 100 mg GL per day) [96]. Therefore, it is crucial to investigate whether 18-GA is still a reliable biomarker when measured after the intake of a low dose of liquorice, or after repeated intake of low doses of liquorice. This is especially important since some studies were unable to measure 18-GA in urine, because the amount was below the detection limit at lower concentrations of intake [51, 54]. Clearly lacking in the literature for all candidate BFIs for liquorice were long-term intervention studies, dietary pattern studies and observational studies. Currently, the longest study had a duration of 4 weeks. However, this study was done using liquorice flavonoid oil in which glycyrrhizin was almost removed from the product (< 0.005%) [56]. Consequently, this study did not give us information about the consumption of solid liquorice or other products using liquorice root extracts, which have a higher glycyrrhizin content. The lack of observational and dietary pattern studies makes it impossible to properly validate any of the three found candidate BFIs; therefore, these studies are urgently needed. Moreover, we only found the polyphenol glabridin as candidate BFI for liquorice intake; however, there are more polyphenols known to be present in liquorice roots. For example, Vaya et al. [97] have isolated hispaglabridin A, hispaglabridin B, 4′-O-methylglabridin, formononetin and glabridin itself from liquorice roots. It should be further examined what the exact contribution of these polyphenols is in liquorice (products) and whether these compounds could be possible BFIs for liquorice (products). For 18-GA and glabridin, commercial standards are available; for 18-GA also, a non-commercial standard is available through FoodComEx (Food Compound Exchange, foodcomex.org), an online catalogue of pure compounds made available by academic laboratories [98].

## Conclusions

In this paper, we have identified potential BFIs for cocoa (products) and liquorice (products). For cocoa (products), none of the individual BFIs were found to be specific. However, a combination of individual BFIs might lead to discriminating profiles between cocoa (products) and foods with a similar composition. This needs to be further explored. We did identify 18-GA as a promising candidate BFI for liquorice; however, important information on its validity is still missing, and therefore, more research is needed. This systematic review shows that there is still an urgent need for research to identify specific and valid biomarkers of the consumption of cocoa (products) and liquorice (products).

## Additional file

Additional file 1: **Table S1.** Overview of the studies on biomarkers for cocoa and chocolate, including information about dosage, study design, number of subjects, method used, sample type and the original references. (DOCX 102 kb)

## Abbreviations

18-GA: 18-Glycyrrhetinic acid
3-MGA: 3β-Monoglucuronyl-18β-glycyrrhetinic acid
BFIRev: Biomarker of Food Intake Reviews
BFIs: Biomarkers of food intake
FooDB: Food DataBase
FoodBAll: Food Biomarkers Alliance
FoodComEx: Food Compound Exchange
HMDB: Human Metabolome DataBase
HOMA-IR: Homeostatic Model Assessment-Insulin Resistance
