# Supplementary material for: Biomarkers of food intake for cocoa and liquorice (products): a systematic review
Source: Genes Nutr. 2018 Jul 27;13:22. doi: 10.1186/s12263-018-0610-x (PMC6062926; doi:10.1186/s12263-018-0610-x)
Supplement: Supplementary file 1 — Table S1. Overview of the studies on biomarkers for cocoa and chocolate, including information about dosage, study design, number of subjects, method used, sample type and the original references. (DOCX 102 kb) [file 12263_2018_610_MOESM1_ESM.docx]

**Additional file 1 Table S1 Overview of the studies on biomarkers for cocoa and chocolate , including information about dosage, study design, number of subjects, method used, sample type and the original references.**

| **Dietary factor** | | **Dose of intervention** | **Study design** | **Number of subjects** | **Analytical method** | **Approach** | **Sample type** | **Candidate Biomarkers of Food Intake** | **Primary Reference** |
| --- | --- | --- | --- | --- | --- | --- | --- | --- | --- |
| **Cocoa (products)** | | | | | | | | |  |
| **Solids** | Dark chocolate | 6 mg/kg/day theobromine | Acute single dose study | 12 | HPLC | Targeted | Blood (0 - 24 h after intake) | Theobromine | [82] |
|  | [8^-14^C] theobromine | 10 mg/kg theobromine sodium acetate (equivalent to 6 mg/kg) with 60 j.LCi [8^-14^C]theobromine |  |  | HPLC-radioactivity monitor | Targeted | Urine (0 - 72 h after intake) | Theobromine  6-Amino-5(N-methylformylamino)-1methyluracil  Methylxanthine  7-methyluric acid  7-methylxanthine  3-methylxanthine |  |
|  | Dark chocolate | 40 g and 80 g with bread and water | Acute single dose study | 8 | HPLC-UV  HPLC-FLD | Targeted | Plasma (0 - 8 h after intake) | (–)-Epicatechin  Theobromine | [71] |
|  | Procyanidin-rich chocolate | 80 g of procyanidin-rich chocolate in the form of 105 g of M&M’s | Acute single dose study | 13 | HPLC-ECD | Targeted | Plasma (0 - 6 h after intake) | (–)-Epicatechin (sum free and conjugated metabolites) | [99] |
|  | Flavanol-rich chocolate | 80 g with bread and water | Acute single dose study | 11 | GC-MS  HPLC-MS/MS | Targeted | Urine (24 h before and 3 - 48h after intake) | 3,4-dihydroxyphenylpropionic acid  m-hydroxyphenylpropionic acid  Ferulic acid  3,4-dihydroxyphenylacetic acid  m-hydroxyphenylacetic acid  Phenylacetic acid  vanillic acid  m-hydroxybenzoic acid  p-hydroxybenzoic acid  p-hydroxyhippuric acid  Hippuric acid | [73] |
|  | Dark chocolate | 100 g | Acute single dose study | 5 | UPLC-MS/MS and LC-MS | Targeted | Urine (5 and 10h after intake) | 3′-O-methyl-(-)-epicatechin-5-*O*-sulphate  3′-O-methyl-(-)-epicatechin-7-*O*-sulphate  4′-O-methyl-(-)-epicatechin-5-*O*-sulphate  4′-O-methyl-(-)-epicatechin-7-*O*-sulphate | [57] |
|  | Dark chocolate | 100 g | Acute single dose study | 5 | UPLC-MS/MS | Targeted | Urine (0 - 24 h after intake) | (-)-epicatechin-3′-β-D-glucuronide  (-)-epicatechin-4′-β-D-glucuronide  (-)-epicatechin-7′-β-D-glucuronide  (-)-epicatechin 3′sulfate  (-)-epicatechin 4′-sulfate  (-)-epicatechin 5-sulfate  3′-*O*-methyl-(-)-epicatechin 4′-sulfate  3′-*O*-methyl-(-)-epicatechin 5-sulfate  3′-*O*-methyl-(-)-epicatechin 7-sulfate  4′-*O*-methyl-(-)-epicatechin 5-sulfate  4′-*O*-methyl-(-)-epicatechin 7-sulfate  3′-*O*-methyl-(-)-epicatechin-β-D-glucuronide (a)  3′-*O*-methyl-(-)-epicatechin-β-D-glucuronide (b)  3′-*O*-methyl-(-)-epicatechin-β-D-glucuronide (c)  4′-*O*-methyl-(-)-epicatechin-β-D-glucuronide (a) | [58] |
|  |  |  |  |  |  |  | Plasma (0 - 24h after intake) | (-)-epicatechin-3′-β-D-glucuronide  (-)-epicatechin-4′-β-D-glucuronide  (-)-epicatechin-7′-β-D-glucuronide  (-)-epicatechin 3′-sulfate  (-)-epicatechin 4′-sulfate  3′-O-methyl-(-)-epicatechin 4′-sulfate  3′-O-methyl-(-)-epicatechin 5-sulfate  3′-O-methyl-(-)-epicatechin 7-sulfate  4′-O-methyl-(-)-epicatechin 5-sulfate  4′-O-methyl-(-)-epicatechin 7-sulfate |  |
|  | Dark chocolate | 50 g/d | Parallel intervention study (1 wk: regular consumers of chocolate vs indifferent consumers) | 20 | ^1^H NMR | Untargeted | Urine (morning spot) | Butyrate  3-Hydroxybutyrate  3-Hydroxyisovalerate  Alanine  4-Cresol sulfate  Trimethylamine  Dimethylglycine  Acylcarnitines  Carnitine  trimethyl-amine-N-oxide  Taurine  Guanidinoacetate  Creatinine  Urea  3-(3-hydroxyphenyl)-propionate  4-hydroxyphenylacetate  Phenylacetate  Phenylacetylglutamine  Indoxyl-sulfate  Hippurate  7-Methylxanthine  Theobromine  Trigonelline | [64] |
|  |  |  |  |  | HPLC-MS | Untargeted |  | 2S-1-(3-4-dihydroxyphenyl)-3-(2-4-6-trihydroxyphenyl)-propan-2-ol methylated sulfate  5-(3,4-dihydroxyphenyl)-valeric-acid glucuronide  5-(3,4-dihydroxyphenyl)-valeric-acid methylated sulfate  5-(3,4-dihydroxyphenyl)-valeric-acid sulfate  5-(3-4-dihydroxyphenyl)-valerolactone  5-(3-4-dihydroxyphenyl)-valerolactone glucuronide  5-(3-4-dihydroxyphenyl)-valerolactone methylated glucuronide  5-(3-4-dihydroxyphenyl)-valerolactone methylated sulfate  5-(3-4-dihydroxyphenyl)-valerolactone sulfate  5-(3-hydroxyphenyl)-valerolactone glucuronide  5-(3-hydroxyphenyl)-valerolactone sulfate  5-(4-hydroxy(3-4-dihydroxyphenyl)-valeric-acid  5-(4-hydroxy(3-4-dihydroxyphenyl)-valeric-acid glucuronide  5-(4-hydroxy(3-4-dihydroxyphenyl)-valeric-acid methylated glucuronide  5-(4-hydroxy(3-4-dihydroxyphenyl)-valeric-acid methylated sulfate  5-(4-hydroxy(3-4-dihydroxyphenyl)-valeric-acid sulfate  epicatechin glucuronide  Methylated epicatechin sulfate |  |
|  | Chocolate bar with different flavanol content and sweeteners | 25 g serving containing 48 mg, 251mg, or 266mg of total cocoa flavanols | Acute crossover study | 15 | HPLC-FLD/UV | Targeted | Plasma (0 - 4 h, after intake) | (-)-Epicatechin  3'-*O*-Methyl-epicatechin  4'-*O*-Methyl-epicatechin | [76] |
| **Drinks** | Cocoa beverage | 75 g of cocoa powder in 500 mL of water | Acute single dose study | 6 | HPLC-FLD | Targeted | 10 mL of gastric sample  (0 and intervals of 10 min until stomach was emptied) | Catechin  (–)-Epicatechin  Proacyanidin B2  Proacyanidin B5  Proacyanidin C1 | [72] |
|  | Flavanol-rich cocoa beverage | 0.375 g cocoa/kg body weight | Acute single dose study | 5 | HPLC-MS/MS | Targeted | Plasma (0 - 6h after intake) | Procyanidin dimer B2  Catechin  Epicatechin | [62] |
|  | Cocoa beverage | 10 g of cocoa powder in 200 ml of water | Acute crossover study | 9 | HPLC-ESI-MS/MS | Targeted | Urine (0 - 24h after intake) | Epicatechin | [63] |
|  | Cocoa beverage | 40 g of cocoa powder in 250 mL of whole milk vs. whole milk only | Acute crossover study | 5 | HPLC-MS/MS | Targeted | Plasma (0 and 2 h after intake) | (–)-Epicatechin sulfates  (–)-Epicatechin glucuronide  (–)-Epicatechin methylglucuronide  (–)-Epicatechin sulfoglucuronide  (–)-Epicatechin methyl sulfoglucuronide | [79] |
|  |  |  |  |  |  |  | Urine (0 and 6 h after intake) | (–)-Epicatechin glucuronide |  |
|  | Flavanol cocoa drinks | Cocoa beverage powder containing 917 mg (high-flavanol cocoa drink) and 37 mg (low-flavanol cocoa drink) of total flavanol in 300 mL of water | Acute crossover study | 10 | HPLC-MS  ^1^H-NMR  ^13^C-NMR | Targeted | Plasma (0 - 6 h after intake) | 4'-*O*-Methyl-epicatechin-7-O-β-D-glucuronide  4'-*O*-Methyl-epicatechin  3'-*O*-Methyl-epicatechin-5/7-O-β-D-glucuronide  3'-*O*-Me-epicatechin  Epicatechin-7-O-β--D-glucuronide  Epicatechin  Catechin  4'-O-Methyl-catechin  3'-O-Methyl-catechin | [81] |
|  | Cocoa beverage | 40 g of cocoa powder in 250 mL of whole milk or water | Acute crossover study | 21 | HPLC-MS/MS | Targeted | Plasma (0 - 6 h after intake) | (–)-Epicatechin-glucuronide | [80] |
|  | Cocoa beverage | 40 g of cocoa powder in 250 mL of whole milk | Acute crossover study | 21 | HPLC-MS/MS | Targeted | Urine (0 - 24 h after intake) | (–)-Epicatechin-glucuronide  (–)-Epicatechin-sulfates | [77] |
|  | Cocoa beverage | 40 g of cocoa powder in 250 mL of whole milk or water | Acute crossover study | 21 | HPLC-MS/MS | Targeted | Urine (0 - 24 h, after intake) | (–)-Epicatechin-glucuronide  (–)-Epicatechin-sulfates | [78] |
|  | Cocoa beverage | 20.3 of alkalized cocoa powder in 400 mL of water | Acute single dose study | 8 | HPLC-MS/MS  NMR | Targeted | Urine (0 - 8 h after intake) | N-[3',4'-dihydroxy-(E)-cinnamoyl]-L-aspartic acid  N-[3',4'-dihydroxy-(E)-cinnamoyl]-L-dopa  N-[3',4'-dihydroxy-(E)-cinnamoyl]-L-tyrosine  N-[4'-hydroxy-(E)-cinnamoyl]-L-aspartic acid  N-[4'-hydroxy-(E)-cinnamoyl]-L-glutamic acid  N-[4'-hydroxy-(E)-cinnamoyl]-L-dopa  N-[4'-hydroxy-(E)-cinnamoyl]-L-tyrosine  N-[4'-hydroxy-3'-methoxy-(E)-cinnamoyl]-L-aspartic acid  N-cinnamoyl-L-aspartic acid  N-[4'-hydroxy-(E)-cinnamoyl]-L-tryptophane  N-[4'-hydroxy-3'-methoxy-(E)-cinnamoyl]-L-tyrosine | [32] |
|  | Cocoa beverage | 40 g of cocoa powder in 250 mL of milk or water | Acute crossover study | 10 | HPLC-qTOF-MS | Untargeted | Urine (baseline, 0 - 24 h after intake) | Tyrosine  Hydroxynicotinic acid  Trigonelline  Hydroxyacetophenone  Vanillic acid  Vanilloylglycine  Cyclo(Pro-Pro)  Epicatechin-O-sulfate  *O*-Methylepicatechin  5-(3′,4′-dihydroxyphenyl)-γ-valerolactone-sulfate  3,5-Diethyl-2-methylpyrazine  Caffeine  6-amino-5-[*N*-methylformylamino]-1-methyluracil  7-methyluric acid  7-methylxanthine  3-methyluric acid  3-methylxanthine  3,7-dimethyluric acid  Theobromine  Cyclo(Ser-Tyr)  4-hydroxy-5-(3,4-dihydroxyphenyl)-valeric acid  5-(3′,4′-dihydroxyphenyl)-γ-valerolactone glucuronide  5-(3′,4′-dihydroxyphenyl)-g-valerolactone glucuronide  3′-methoxy-4′-hydroxyphenylvalerolactone glucuronide  3′-methoxy-4′-hydroxyphenylvalerolactone | [44] |
|  | Cocoa beverage | 10 g of green and black’s organic cocoa in 250 ml of milk or water | Acute crossover study | 9 | HPLC-PDA-MS/MS | Targeted | Urine (0 - 24 h after intake) | (epi)catechin-O-sulfate,  (-)-Epicatechin-*O*-glucuronide,  *O*-Methyl-(epi)catechin-*O*-sulfate | [66] |
|  |  |  |  |  |  |  | Plasma (0 - 24h after intake) | (epi)catechin-O-sulfate,  *O*-Methyl-(epi)catechin-*O*-sulfate |  |
|  | Cocoa beverage | 40 g of cocoa powder in 250 mL of water for humans, and 4.8 g natural cocoa powder/kg/day for rats | Acute crossover study | 21 | HPLC-MS/MS | Targeted | Urine (0 and 24 h after intake) | 3,4-butyrate acid  m-Coumaric acid  p-Coumaric acid  Caffeic acid  Ferulic acid  3,4-Dihydroxyphenylacetic acid  3-Methoxy-4-hydroxyphenylacetic acid  3-Hydroxyphenylacetic  Phenylacetic acid  Protocatechuic acid  Vanillic acid  4-Hydroxybenzoic acid  3-Hydroxybenzoic acid  4-Hydroxyhippuric acid  Hippuric acid;  Enterodiol;  Enterolactone;  (−)-Epicatechin;  Procyanidin B2;  5-(3′,4′-dihydroxyphenyl)-γ-valerolactone  5-(3′-methoxy,4′-hydroxyphenyl)-γ-valerolactone | [84] |
|  | Cocoa beverage | 40 g of cocoa powder in 500 mL of skimmed milk per day | Crossover Intervention study (4 wk of control beverage and 4 wk of cocoa beverage) | 42 | HPLC-MS/MS | Targeted | Urine (baseline and 24 h, after intervention) | Epicatechin-O-glucuronides;  Epicatechin-O-sulfates;  O-Methyl-epicatechin-O-glucuronides;  O- Methyl-epicatechin-O-sulfates;  5-(3′,4′-dihydroxyphenyl)-γ-valerolactone-O-glucuronides  5-(3′,4′-dihydroxyphenyl)-γ-valerolactone-O-sulfates  5-(3′-methoxy,4′-hydroxyphenyl)-γ-valerolactone-O-glucuronides  5-(3′-methoxy,4′-hydroxyphenyl)-γ-valerolactone-O-sulfates  (−)-Epicatechin  5-(3′,4′-dihydroxyphenyl)-γ-valerolactone  3,4-Dihydroxyphenylacetic acid  3-Hydroxyphenylacetic acid  Vanillic acid | [85] |
|  |  |  |  |  |  |  | Plasma (baseline and 24 h, after intervention) | 5-(3′,4′-dihydroxyphenyl)-γ-valerolactone-*O*-glucuronides  5-(3′-methoxy,4′-hydroxyphenyl)-γ-valerolactone-*O*-glucuronides  5-(3′,4′-dihydroxyphenyl)-γ-valerolactone  3,4-Dihydroxyphenylacetic acid |  |
|  | Cocoa beverage | 40 g of cocoa powder in 250 mL of milk | Acute single dose study | 10 | HPLC-qTOF-MS | Untargeted | Urine (before (-2 and 0 h) and 2 - 24 h after intake) | N-methylguanine  Vanilloylglycine  Dihydroxyphenyl valerolactone glucuronide  Furoylglycine  7-methylxanthine  3-methylxanthine  Theobromine  Xanthurenic acid | [34] |
|  | Cocoa beverage | 40 g of cocoa powder in 250 mL of milk or water | Acute crossover study | 21 | HPLC-MS/MS | Targeted | Urine (0 - 24 h after intake) | 3,4-Dihydroxyphenylpropionic acid  m-Coumaric acid  p-Coumaric acid  Caffeic acid  Ferulic acid  3,4-Dihydroxyphenylacetic acid  3-Methoxy-4-hydroxyphenylacetic acid  3-Hydroxyphenylacetic  Phenylacetic acid  Protocatechuic acid  Vanillic acid  4-Hydroxybenzoic acid  3-Hydroxybenzoic acid  4-Hydroxyhippuric acid  Hippuric acid | [83] |
|  | Cocoa beverage | 46 g of cocoa powder in 250 mL of whole milk | Acute single dose study | 1 | HPLC-CEAD | Targeted | Plasma (0 and 2 h after intake) | (–)-Catechin*  (+)-Catechin* | [74] |
|  | Cocoa beverage | 0.5 g/kg bw low flavanol cocoa powder, 5 g/kg bw milk (1% fat), and 22 mg/kg bw cocoa extract containing 47.7% (w/w) flavanols | Acute single dose study | 10 | HPLC-MS/MS | Targeted | Plasma (0 - 4 h, after intake) | (–)-Epicatechin  (–)-Epicatechin-3'-β-D-glucuronide  (–)-Epicatechin-3'-sulfate  (–)-Epicatechin-5-sulfate  (–)-Epicatechin -7-sulfate  3'-*O*-Methyl-epicatechin-5/7-sulfates  4'-*O*-Methyl-epicatechin-5/7-sulfates  4'-*O*-Methyl-epicatechin-7-β-D-glucuronide  3′-*O*- Methyl-epicatechin*  4′-*O*- Methyl-epicatechin* | [69] |
|  | Cocoa beverage | 40 g/d of cocoa powder in 500 mL of skimmed milk or only skimmed milk | Crossover intervention study (4 wk of control beverage and 4 wk of cocoa beverage) | 20 | HPLC-qTOF-MS | Untargeted | Urine (baseline and 24h after intervention) | Hydroxynicotinic acid  7-methyluric acid  Tyrosine sulfate  3-methyluric acid  Butyrylcarnitine  7-methylxanthine  Methylglutarylcarnitine  3-methylxanthine  3,7-dimethyluric acid  Cyclo(propylalanyl)  3,5-diethyl-2-methylpyrazine  Theobromine  Vanillic acid glucuronide  Vanilloylglycine  4-hydroxy-5-(dihydroxyphenyl)-valeric acid glucuronide  3′-methoxy-4′-hydroxyphenylvalerolactone  4-hydroxy-5-(hydroxy-methoxyphenyl)-valeric acid glucuronide  N-[4′-hydroxycinnamoyl]-l-aspartic acid  5-(3′,4′-dihydroxyphenyl)-γ-valerolactone glucuronide  (Epi)catechin glucuronide  Methoxyhydroxyphenylvalerolactone glucuronide  N-[4′-hydroxy-3′-methoxy-E-cinnamoyl]-l-aspartic acid  5-(3′,4′-dihydroxyphenyl)-γ-valerolactone sulfoglucuronide  5-(3′,4′-dihydroxyphenyl)-γ-valerolactone-sulfate  Hydroxyphenyl-γ-valerolactone glucuronide  Vanillic acid sulfoglucuronide,  4-hydroxy-5-(dihydroxyphenyl)-valeric acid sulfate  Epicatechin sulfoglucuronide  Methyl-(epi)catechin sulfate  Hydroxyphenylvalerolactone sulfate  5-(hydroxy-methoxy-phenyl)-γ-valerolactone sulfate  4-hydroxy-5-(phenyl)-valeric acid sulfate | [33] |
|  | Cocoa beverage | 15 g of cocoa powder (control) and 25 g of cocoa powder enriched in methylxanthines, in 200 mL of semi-skimmed milk | Acute crossover study | 13 | LC-QTOF and LC-DAD | Targeted | Urine (baseline (-2-0h), 0 - 24 h after intake) | 1-methylxanthine  3-methylxanthine  7-methylxanthine  1,7-dimethylxanthine  3,7-dimethylxanthine  1,3-dimethylxanthine  1,3,7-trimethylxanthine  1-monomethyluric acid  1,3-dimethyluric acid  1,7-dimethyluric acid  3,7-dimethyluric acid  1,3,7-trimethyluric acid | [65] |
|  |  |  |  |  | HPLC-DAD | Targeted | Plasma (0 - 8h after intake) | Caffeine  Paraxanthine  Theobromine  Theophylline  3-methylxanthine  7-methylxanthine |  |
|  | Fruit-flavored cocoa powder beverage | Cocoa powder containing 5.3 mg and 10.7 of cocoa-flavanol/kg BW in 500 mL of water | Acute crossover study | 40 | HPLC-FLD-UV-ECD | Targeted | Plasma (0-24 h, after intake) | Epicatechin-3'-β-D-glucuronide  Epicatechin-3'-sulfate  3'-*O*-Methyl-epicatechin-5-sulfate  3'-*O*-Methyl-epicatechin-7-sulfate  (–)-Epicatechin  Epicatechin-5-sulfate  Epicatechin-7-sulfate | [75] |
|  |  |  |  |  |  |  | Urine (0 - 24 h after intake) | Epicatechin-3'-β-D-glucuronide  Epicatechin-3'-sulfate  3'-*O*-Methyl-epicatechin-5-sulfate  3'-*O*-Methyl-epicatechin-7-sulfate  5-(3,4-dihydroxyphenyl)-L-valerolactone* |  |
| **Cocoa products** | Average Danish Diet (including chocolate) and. New Nordic Diet | ADD contained cocoa products, NDD free of cocoa products | Parallel intervention study (6 months) | 181 | UPLC-qTOF-MS | Untargeted | Urine (24h in week 0, 4, 12, 20 and 26) | Theobromine  7-methyluric acid  6-amino-5-[N-methylformylamino]-1-methyluracil  3,7-dimethyluric acid  7-methylxanthine | [60] |
|  | Average Danish Diet (including chocolate) vs. New Nordic Diet | ADD contained cocoa products, NDD free of cocoa products | Parallel intervention study (6 months) | 107 | UPLC-qTOF-MS | Untargeted | Urine (24h in week 0, 12 and 26) | Theobromine,  7-methyluric acid  6-amino-5-[N-methylformylamino]-1-methyluracil | [59] |
|  | Cocoa-containing food items | Based on (24h-dietary recall) | Observational study  Cross-sectional | 481 | UPLC-qTOF-MS | Untargeted | Urine (24h-collection) | Methyl(epi)catechin sulfate  Vanillic acid sulfate | [26] |
|  | Cocoa-containing food items | Based on FFQ  (non-consumers vs. consumers) | Observational study  Cross-sectional | 64 | HPLC-qTOF-MS | Untargeted | Spot urine | Xanthine  6-amino-5[N-methylformylamino]-1-methyluracil  6-amino-5[N-methylformylamino]-1-methyluracil isomer  3-methyluric acid  7-methylxanthine  3- methylxanthine  3,7-dimethyluric acid  Theobromine  Furoglycine  cyclo(aspartyl-phenylalanyl  Aspartyl-phenylalanine  Vanillin sulphate  (Epi)catechin glycuronide  Vanillic acid  (Epi)catechin sulphate  4-hydroxy-5-(dihydroxyphenyl)valeric acid glucuronide  4-hydroxy-5-(hydroxy-methoxyphenyl)-valeric acid glucuronide  Methoxyhydroxyphenylvalerolactone,  5-(3′,4′-Dihydroxyphenyl)-valerolacetone sulfoglucuronide  5-(3′,4′-Dihydroxyphenyl)-valerolacetone glucuronide  4-hydroxy-5-(dihydroxyphenyl)valeric acid  4-hydroxy-5-(dihydroxyphenyl)valeric acid sulphate  5-(3′,4′-Dihydroxyphenyl)-valerolacetone glucuronide  4-hydroxy-5-(hydroxymethodyphenyl)-valeric acid sulphate  Methoxyhydroxyphenylvalerolactone glucuronide  Hydroxyphenyl-valeroacetone glucuronide  5-(3′,4′-Dihydroxyphenyl)-valerolacetone sulphate  Hydroxyphenyl-valeroacetone sulphate  4-hydroxy-5-(hydroxyphenyl)-valeric acid sulphate  4-hydroxy-5-(phenyl)-valeric acid sulphate  Methylglutarylcarnitine | [27] |
| **Miscellaneous** | Chocolate and cocoa | 96 g of chocolate and  66 g cocoa | Acute crossover study | 5 | HPLC-MS | Targeted | Urine (0-24 h after intake) | Epicatechin (non)methylated -free/glucuronide/sulfate/sulfoglucuronide  Methylepicatechin- sulfoglucuronide | [61] |
|  |  |  |  |  |  |  | Plasma (0- 24 h after intake) |  |  |
|  | Chocolate bars and cocoa beverages | 40 g bars and 250 mL beverages | Acute crossover study | 6 | RP-HPLC-ECD | Targeted | Serum (0 - 6 h after intake) | (±)-Catechin  (–)-Epicatechin | [67] |
|  | Cocoa in nut cream and capsules | Capsules: cocoa polyphenol extract in high-amylose maize starch 1:9 (w/w)  Cocoa-nut creams: 20% (w/w) cocoa for control cream, and 1·5% of free or encapsulated cocoa polyphenol extract | Acute crossover study | 12 | HPLC-MS/MS | Targeted | Serum (0 - 24 h after intake) | Chlorogenic acid  Caffeic acid  Hippuric acid  Hydroxybenzoic acid  4-hydroxyphenylacetic acid  3-(4-hydroxyphenyl)propionic acid  5-(3′,4′,-dihydroxyphenyl)- γ -valerolactone | [86] |
|  |  |  |  |  |  |  | Urine (0 - 24 h after intake) | (epi)catechin  Epigallocatechin  Procyanidin  Protocatechuic acid  Vanillic acid  Ferulic acid  Caffeic acid  Coumaric acid  Chlorogenic acid  Hippuric acid  Homovanillic acid  Hydroxybenzoic acid  3,4-dihydroxyphenylacetic acid  Dihydrocaffeic acid  Dihydroferulic acid  3-(4-hydroxyphenyl)propionic acid  4-hydroxyphenylacetic acid  5-(3′,4′,-dihydroxyphenyl)- γ -valerolactone |  |
|  |  |  |  |  |  |  | Faeces (on the day of experiment) | (Epi)catechin  Epigallocatechin  Procyanidin  Protocatechuic acid  Vanillic acid  Ferulic acid  Caffeic acid  Chlorogenic acid  Homovanillic acid  Dihydrocaffeic acid  Dihydroferulic acid  3-(4-hydroxyphenyl)propionic acid  5-(3`,4`,-dihydroxyphenyl)- γ -valerolactone |  |
|  | Cocoa flavanols-capsules | Part 1: Cocoa flavanol extract:  1000 mg/d (2 capsules/d in wk 1+2), 1500 mg/d (3 capsules/d in wk 3+4), and 2000 mg/d (4 capsules/d in wk 5+6)  Part 2: Cocoa flavanol extract:  1000 mg/d (2 capsules/d in wk 1), 1500 mg/d (3 capsules/ d in wk 2), and 2000 mg/d (4 capsules/d in wk 3-10) | Crossover intervention study (Open-label, intake-amount escalated, 6 wk)  Single arm Intervention study (12 wk) | 33 | HPLC-UV/Vis-FLD-ECD  HPLC-MS/MS | Targeted | Plasma (0-2 h, after intake and on days 1,15,29,43, and 57 of the intervention) | Ec-3'-β-D-glucuronide  Ec-3'-sulfate  3'-*O*-Methyl-epicatechin-5-sulfate  3'-*O*-Methyl-epicatechin-7-sulfate  4′-*O*-Methyl(−)-epicatechin-5-sulfate  4′-*O*-Methyl(−)-epicatechin-7-sulfate  (–)-Epicatechin  5-(3',4'-dihydroxyphenyl)- γ -valerolactone  Theobromine  Caffeine  Paraxanthine | [68] |
|  |  |  |  | 59 |  | Targeted | Plasma (0-2 h, after intake and on days 1,43,85, and 99 of the intervention) | 5-(3',4'-dihydroxyphenyl)- γ –valerolactone  Theobromine |  |
|  | Cocoa extract as part of a ready-to-eat meals | 1.4 gram cocoa extract | Single arm Intervention study (4 wk, part of a weight loss diet) | 47 | HPLC-qTOF-MS | Untargeted | Urine (24h, before and after the study) | 3-methylxanthine  3-methyluric acid  l-Beta-Aspartyl-L-phenylalanine  2,5,7,3’,4’-Penthahydroxyflavanone 5-O-glucoside  7,4’-Dimethoxy-6-C­-methylflavanone  3-methoxy-4-hydroxyphenylglycol sulphate  Uridine monophosphate | [100] |

Abbreviations: ^1^H NMR, Proton nuclear magnetic resonance; ^13^C NMR, Carbon-13 (C13)nuclear magnetic resonance; CEAD, coulometric electrode array detector; DAD, diode array detector; ECD, Electrochemical detection; ESI, Electrospray ionization; FLD, fluorescence detector; GC-MS, Gas Chromatography - Mass spectrometry; GL, glycyrrhizin; HPLC, High-performance liquid chromatography; LC, liquid chromatography; MS/MS, Liquid chromatography-tandem mass spectrometry; PDA, photodiode array detector; RP-HPLC, Reversed-phase high-performance liquid chromatography; SPE, solid phase extraction liquid chromatography tandem mass spectrometry; UPLC: Ultra performance liquid chromatography; UV, Ultraviolet ; VIS, visible light
